# ESCAIDE ‘late-breaker’ abstract call opens 1 September 2016

**DOI:** 10.2807/1560-7917.ES.2016.21.45.30322

**Published:** 2016-08-25

**Authors:** Eurosurveillance editorial team

**Affiliations:** 1 European Centre for Disease Prevention and Control (ECDC), Stockholm, Sweden

The 2016 European Scientific Conference on Applied Infectious Disease Epidemiology (ESCAIDE) will take place from 28 to 30 November 2016.

The later breaker call for abstracts will open on 1 September and may only be submitted online, through the ESCAIDE booking site.

Late breaker abstracts may be eligible for both oral and poster presentation at the Conference. To be eligible, the abstract must fulfil all the following criteria:

a) report on acute urgent public health problems OR b) contain novel, surprising findings; AND
report data or information that was unavailable before 11 May 2016 (the deadline for submission in the general call for abstracts for ESCAIDE), AND
have not been published before.

More information about the late breaker call is available here.

The programme and conference registration instructions are available on the ESCAIDE conference website. For further information, contact: escaide.conference@ecdc.europa.eu

